# Integrative metabolomic and transcriptomic analyses reveals the accumulation patterns of key metabolites associated with flavonoids and terpenoids of *Gynostemma pentaphyllum* (Thunb.) Makino

**DOI:** 10.1038/s41598-024-57716-5

**Published:** 2024-04-15

**Authors:** Xiaomeng Zhao, Weiwei Ge, Zhi Miao

**Affiliations:** School of Chemical Engineering and Technology, Tianjin University, Tianjin, 300350 People’s Republic of China

**Keywords:** *Gynostemma pentaphyllum*, Flavonoid metabolism, Terpenoid metabolism, Transcriptome, Metabolome, Genetics, Plant sciences

## Abstract

*Gynostemma pentaphyllum* (Thunb.) Makino (*G. pentaphyllum*) is a medicinal and edible plant with multiple functions of liver protection, anti-tumor, anti-inflammation, balancing blood sugar and blood lipids. The nutritional value of the *G. pentaphyllum* plant is mainly due to its rich variety of biologically active substances, such as flavonoids, terpenes and polysaccharides. In this study, we performed a comprehensive analysis combining metabolomics and root, stem and leaf transcriptomic data of *G. pentaphyllum*. We used transcriptomics and metabolomics data to construct a dynamic regulatory network diagram of *G. pentaphyllum* flavonoids and terpenoids, and screened the transcription factors involved in flavonoids and terpenoids, including basic helix-loop-helix (bHLH), myb-related, WRKY, AP2/ERF. Transcriptome analysis results showed that among the DEGs related to the synthesis of flavonoids and terpenoids, dihydroflavonol 4-reductase (DFR) and geranylgeranyl diphosphate synthases (GGPPS) were core genes. This study presents a dynamic image of gene expression in different tissues of *G. pentaphyllum*, elucidating the key genes and metabolites of flavonoids and terpenoids. This study is beneficial to a deeper understanding of the medicinal plants of *G. pentaphyllum*, and also provides a scientific basis for further regulatory mechanisms of plant natural product synthesis pathways and drug development.

## Introduction

*Gynostemma pentaphyllum* (Thunb.) Makino (*G. pentaphyllum*) is a cucurbit herb vine widely distributed in South and East Asia^1^. The nutritional value of *G. pentaphyllum* is mainly due to its various bioactive substances, such as flavonoids, terpenoids and polysaccharides^2–5^. Therefore, *G. pentaphyllum* is a plant with the same origin as medicine and food. *G. pentaphyllum* has many functions such as liver protection, anti-tumor, anti-inflammation, and balancing blood sugar and blood lipids^6–10^ that is often made into *G. pentaphyllum* tea edible.

Flavonoids and terpenoids are natural compounds with specific structural characteristics that play important roles in plant growth and development, such as defense, attracting pollinators, and attracting natural enemies^11,12^. In addition to their key roles in plants, flavonoids and terpenoids also have many benefits for human health. Many studies have shown that flavonoids have antioxidant^13^, anti-tumor^14^, cardiovascular health and immune function enhancement effects^15^, while terpenoids have anti-cancer^16^, anti-aging^17^, neuroprotection^18^ and other pharmacological activities^19^. *G. pentaphyllum* is rich in various flavonoids and terpenoids^20^, among which flavonoids are mainly concentrated in the leaves of *G. pentaphyllum*, and more than 200 kinds of terpenoids of gypenoside have been successfully isolated from *G. pentaphyllum*^21^. In comparison to nutritionally rich ginseng, *G. pentaphyllum* has a shorter growth cycle and faster growth rate^22^, with higher concentrations of flavonoids and terpenoids.

With the development of sequencing technology and the reduction of sequencing costs, transcriptome and genome technologies have begun to be applied in the study of medicinal plants^23–25^. A transcriptome study of *Panax notoginseng* revealed 270 genes involved in the triterpenoid saponin biosynthesis in *Panax notoginseng*^26^, and through a comparison of the transcriptome of leaves, roots and flowers found that *CYP716A53v2* gene was highly expressed in the root tissue to participate in triterpene saponin biosynthesis. However, transcriptome and genome data are only used to study medicinal plants from the aspect of gene function without considering the regulatory relationship of metabolites, which is insufficient to comprehensively understand the synthetic network of organisms^27^. Metabolomics^28^ is often used to analyze endogenous small molecule metabolites, and can be combined with other omics tools to effectively elucidate gene functions, metabolite biosynthesis pathways and regulatory mechanisms^29^. The combination of transcriptome and metabolome has been widely used in the study of various medicinal plants, such as the regulation of nitrogen in rice root growth^30^ and the metabolism of anthocyanins^31^. Furthermore, utilizing data obtained from RNA sequencing (RNA-seq) or metabolomics assays to generate modules that rely on weighted gene co-expression network analysis (WGCNA) can narrow down the list of potentially key genes or metabolites^32^. This promising approach has proven effective in identifying modules of co-expressed genes or metabolites and correlating these distinct modules with phenotypic features, further examining key genes (metabolites) in the network and understanding their role in Role in regulatory mechanisms of biological systems.

In this study, we used RNA-Seq combined with ultra-high performance liquid chromatography-time-of-flight mass spectrometry (UHPLC-QTOF-MS) was used to obtain transcriptomic and metabolomic data of different tissues (roots, stems, leaves) of *G. pentaphyllum* for subsequent analysis. And we used these data to screen core genes and metabolites associated with flavonoids and terpenoids by WGCNA, differential expression gene analysis and correlation analysis. In addition, we delve into the interactions between gene transcription levels and the accumulation of metabolites, providing a detailed framework for the actual correlations and differences between the transcriptome and metabolome. The research results show that the tissue of *G. pentaphyllum* leaves has strong ability to synthesize flavonoids, and the terpenoids show dynamic changes in different tissue parts. This lays the foundation for further research on metabolic regulation, synthetic biology and molecular breeding of *G. pentaphyllum*.

## Materials and methods

### Plant material

In this study, the three samples were collected from the *G. pentaphyllum* planting base in Pingli County, Shaanxi Province. These collected samples were identified as *G. pentaphyllum* by Professor Zhi miao. We divided the three *G. pentaphyllum* samples into three groups according to root, stem, and leaf tissues: S1, S2, and S3. The freshly collected materials were promptly frozen in liquid nitrogen and stored at − 80 °C for subsequent analysis. The plants collected in this study and the subsequent experiments performed were in line with the IUCN Policy Statement on Research Involving Species at Risk of Extinction and the Convention on the study Trade in Endangered Species of Wild Fauna and Flora.

### RNA sequencing and analysis

#### RNA sequencing and assembling

In this study, we used the Illumina® NEBNext® Ultra™ RNA Library Prep Kit (NEB, USA) to extract total RNA from root, stem, and leaf t of *G. pentaphyllum*. We prepared a cDNA library from the nine RNA samples according to the method published by Foucart^33^. These cDNA libraries followed the manufacturer's instructions and sequenced on the Illumina HiSeq 2500 high throughput sequencing platform (Illumina, San Diego, CA, USA). And raw sequencing data were uploaded to the NCBI database (Project number: PRJNA1054609) (Table 1). The Trinity method (Parameter: min_kmer_cov: 2, min_contig_length: 200, trimmomatic) was used to assemble high-quality reads into unigenes^34^.

**Table 1:** SRA number from the *G. pentaphyllum* planting for nine samples.

Location	Sample	BioSample	SRA number
Root	S1-1	SAMN38927480	SRS19931191
	S1-2	SAMN38927481	SRS19931192
	S1-3	SAMN38927482	SRS19931193
Stem	S2-1	SAMN38927483	SRS19931194
	S2-2	SAMN38927484	SRS19931195
	S2-3	SAMN38927485	SRS19931196
Leaf	S3-1	SAMN38927486	SRS19931197
	S3-2	SAMN38927487	SRS19931198
	S3-3	SAMN38927488	SRS19931199

In order to obtain real protein coding genes from the assembled unigenes, we compared and annotated these unigenes using a large number of protein functional databases by blast^35^ with the value of 10^−5^, including Cluster of Protein Homology Groups (KOG/COG/eggNOG)^36^, Human Annotated and Reviewed Protein Sequence Database (Swiss-Prot)^37^, Protein Families (Pfam)^38^, Kyoto Encyclopedia of Genes and Genomes (KEGG)^39,40^, NCBI Non-redundant Protein Sequences (NR)^41^, and Gene Ontology (GO)^42^.

#### Identification of differentially expressed genes (DEGs) and statistical analysis

This study used RSEM^43^ (Reference: Protein coding genes from the *G. pentaphyllum* genome, Sequence alignment software: bowtie2) to calculate the gene expression FPKM (Fragments Per Kilobase of transcript per Million mapped reads) of 9 transcriptome gene sequences to explore the differences in gene expression in the roots, stems and leaves of *G. pentaphyllum*. According to the gene expression levels. We analyzed the correlation coefficients of the expression levels of 9 transcriptomes to analyze whether the gene expression in different tissues of *G. pentaphyllum* has the same expression pattern. And then we used the R package DESeq (version 1.10.1) to perform differential expression analysis among different groups in *G. pentaphyllum*. During this analysis, we used the Benjamini–hochberg method^44^ to correct P values. Genes identified by DESeq with a P value of 0.05 were determined as differentially expressed genes (DEGs). Subsequently, we conducted differential expression analysis among distinct groups in *G. pentaphyllum* using the R package DESeq^45^ (version 1.10.1). Throughout this analysis, the Benjamini–Hochberg method^44^ was employed for the correction of P values (*P* < 0.05). We further selected these DEGs for GO enrichment and KEGG analysis^46^ to gain in-depth undeírstanding of their functions and metabolic pathways involved.

We then selected genes related to the synthesis of flavonoids and terpenoids from these DEG-related genes. To further reveal the protein–protein interactions (PPI) of genes involved in the synthesis of flavonoids and terpenoids, we used the STRING database (http://string-db.org/)^47^ to obtain the corresponding data and visualized these interactions in Cytoscape^48^.

### WGCNA related genes networks

In order to recognize the WGCNA modules relevant to terpenoid biosynthesis and flavonoid biosynthesis in various tissues of *G. pentaphyllum*, we constructed a co-expression network with the results of total flavonoids and terpenoids in *G. pentaphyllum* and RNA-seq dataset. We used the R package WGCNA^49^ for WGCNA analysis to identify genes involved in terpenoid and flavonoid biosynthesis, and the WGCNA network construction and module detection were performed utilizing an unsigned topological overlap matrix, soft-thresholding powers set to 30 (genes) and 14 (proteins), a minimum module size of 20, and a branch merge cut height of 0.25^50^. The characteristic gene values of different modules were calculated to evaluate their association with terpenoid and flavonoids abundance in stems and leaves to find genes related to the synthesis of terpenes and flavonoids. And we further selected these WGCNA network for GO enrichment and KEGG analysis to gain in-depth understanding of their functions and metabolic pathways involved.

### Metabolome detection and analysis

The samples of this study were placed in a mortar, added with liquid nitrogen and ground into powdered samples. Then put 60 mg of the powder into an EP tube and added 500 μl of 70% methanol aqueous solution with waiting for 10 min. After filtration with organic filter, the sample was dried under vacuum and room temperature. Finally, 100 ul of methanol aqueous solution was added, vortexed, and the sample was filtered using a 0.2 um organic filter. Prepared samples were detected using UHPLC-QTOF-MS (THERMO UltiMate 3000)^51^ combined with high-resolution mass spectrometry (AB SCIEX 5600 QTOF). The column temperature was set as 40 °C and loading volume was 5 μl. The mass spectrometer (AB 5600 Triple TOF) is controlled by the software (Analyst TF 1.7 AB Sciex) and performs primary and secondary mass spectrometry data acquisition based on IDA function. For the MS data collection, the molecular ions with the highest intensity and more than 100 were selected. The first-order collection range is 50–1200, the bombardment energy: 30 eV and 10 s-order spectra are taken every 50 ms.

Using the MSDIAL software (Parameter: Accurate mass tolerance (MS1): 0.01 Da, Accurate mass tolerance (MS2): 0.05 Da, Identification score cut off: 60%)^52^ was used to perform data processing. Meanwhile, the databases of MassBank^53^, Metlin^54^, MoNA^55^ and HMDB are independently integrated based on the primary and secondary maps, and blank samples are subtracted to get identification results. And we performed multivariate analysis using variable importance projection (VIP) in the orthogonal partial least squares discriminant analysis (OPLS-DA) model^56^ to screen out differentially accumulated metabolites (DAMs). These DAMs were identified as having criteria of VIP value ≥ 1, difference multiple ≥ 2, or ≤ 0.5. Subsequently, k-means clustering and associated heat map analysis were generated in the R environment to gain a more comprehensive understanding of the distribution and trends of DAM.

### WGCNA related metabolic networks

In order to further analyze the WGCNA modules related to terpene biosynthesis and flavonoid biosynthesis in various tissues of *G. pentaphyllum*, this study constructed a co-expression network using the results of total flavonoids and terpenoids in *G. pentaphyllum* and the metabolome dataset. And we used R-package WGCNA^49^ for WGCNA analysis to identify metabolites associated with the biosynthesis of terpenes and flavonoids. We calculated the eigenvalues of different modules to evaluate the relationship between each module and the abundance of terpenoids and flavonoids in *G. pentaphyllum*, looking for metabolites related to the synthesis of terpenes and flavonoids.

### Correlation analysis of transcriptome and metabolome data

We used Pearson correlation test to analyze the correlation between differentially expressed genes (DEGs) and differentially accumulated metabolites (DAMs). We only detected correlations with variable selected correlations with Pearson correlation coefficient (PCC) values ≥ 0.8 and *P* ≤ 0.05. By using “ggplot2” and “getopt” in R, we displayed DEGs and DAMs with PCC ≥ 0.8 between groups in the form of nine-quadrant plots. This graph visually displays the correlation between DEGs and DAMs, with the PCC threshold selected to emphasize highly correlated relationships. Furthermore, we mapped these highly correlated DEGs and DAMs into the KEGG pathway database to predict the metabolic pathways and biological functions they may jointly participate in.

### Validation by qRT-PCR analysis

A total of 20 candidate genes related to flavonoid and terpenoids anabolism were screened for qRT-PCR assay. All primer pairs of these genes were designed using Primer 5.0 (Premier Biosoft, USA). And qRT-PCR was carried out on a LightCycler 96 (F. Hoffmann-La Roche Ltd, Switzerland).

## Result

### RNA-Seq results

In this study, 9 transcriptome data of root, stem and leaf tissues of *G. pentaphyllum* were obtained, and all the original transcriptome data totaled 59.93 Gb. The GC content of the nine transcriptome raw data was 42–45% (Table S1), while the Q30 value of all the transcriptome data is above 92.47%, indicating that the data of this study can be further analyzed. After the assembly and screening of these raw transcripts, a total of 94,850 unigenes were obtained in this study with an average length of 1351 bp and an N50 of 1706 bp, of which 20.97% were longer than 2 kb (Table S2).

In order to further analyze the functions of the assembled unigenes, we used 8 databases to annotate the assembled transcriptome of *G. pentaphyllum*. We annotated a total of 79,280 items in the 8 databases, accounting for 83.5% of the total (Table S3). Among them, the NR database has the most annotated genes, accounting for 78.10%.

### DEGs Identification and enrichment analyses

**Figure 1:**
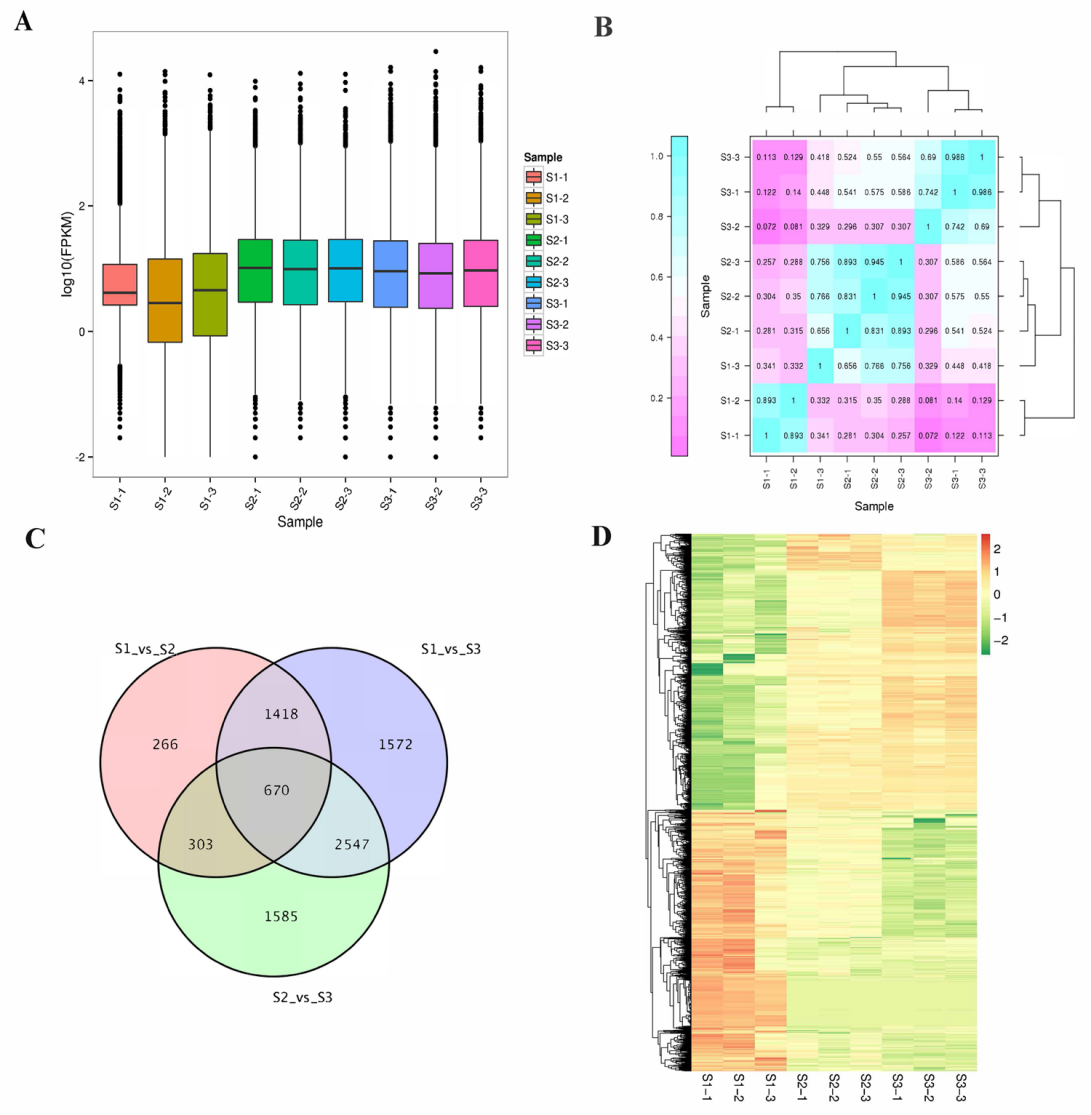
Gene expression levels and DEGs of nine *G. pentaphyllum* transcriptomes. (**A**) Box map of gene expression levels for nine transcriptomes. (**B**) Correlation coefficients of gene expression levels across nine transcriptomes. (**C**) Vane plots for differentially expressed genes (DEGs) among samples S1 (root), S2 (stem), and S3 (leaf). (**D**) Expression levels of DEGs in different samples.

We calculated the gene expression levels of three different tissues of *G. pentaphyllum* through RSEM, and the analysis results showed that the gene expression levels in the stems and leaves of were similar, while the gene expression levels in the roots were different (Fig. 1A). In order to further explore whether the gene expression patterns of *G. pentaphyllum* in different tissues are different, we conducted correlation analysis on the gene expression levels of these 9 transcriptomes. The correlation coefficient of *G. pentaphyllum* in root-to-root was less than 0.5 (Fig. 1B), while the correlation coefficient of stem-to-stem and leaf-to-leaf was greater than 0.5. This further proves that different individuals have different expression patterns of the root tissue genes of *G. pentaphyllum* which may be related to its growth and development.

We identified a total of 8361 DEGs in the three group tissues of *G. pentaphyllum* transcriptome (Fig. 1C). There are 2657 DEGs in root and stem (S1 vs S2), including 1118 up-regulated genes and 1539 down-regulated genes. There were 6207 DEGs in root and leaf (S1 vs. S3), including 2826 up-regulated genes and 3381 down-regulated genes. Stem and leaf (S2 vs. S3) had 5105 DEGs, including 2497 up-regulated genes and 2608 down-regulated genes. We then further analyzed DEGs gene expression in S1, S2 and S3 (Fig. 1D), and the results showed that the level of gene expression in root tissues was significantly different from that in stems and leaves.

**Figure 2:**
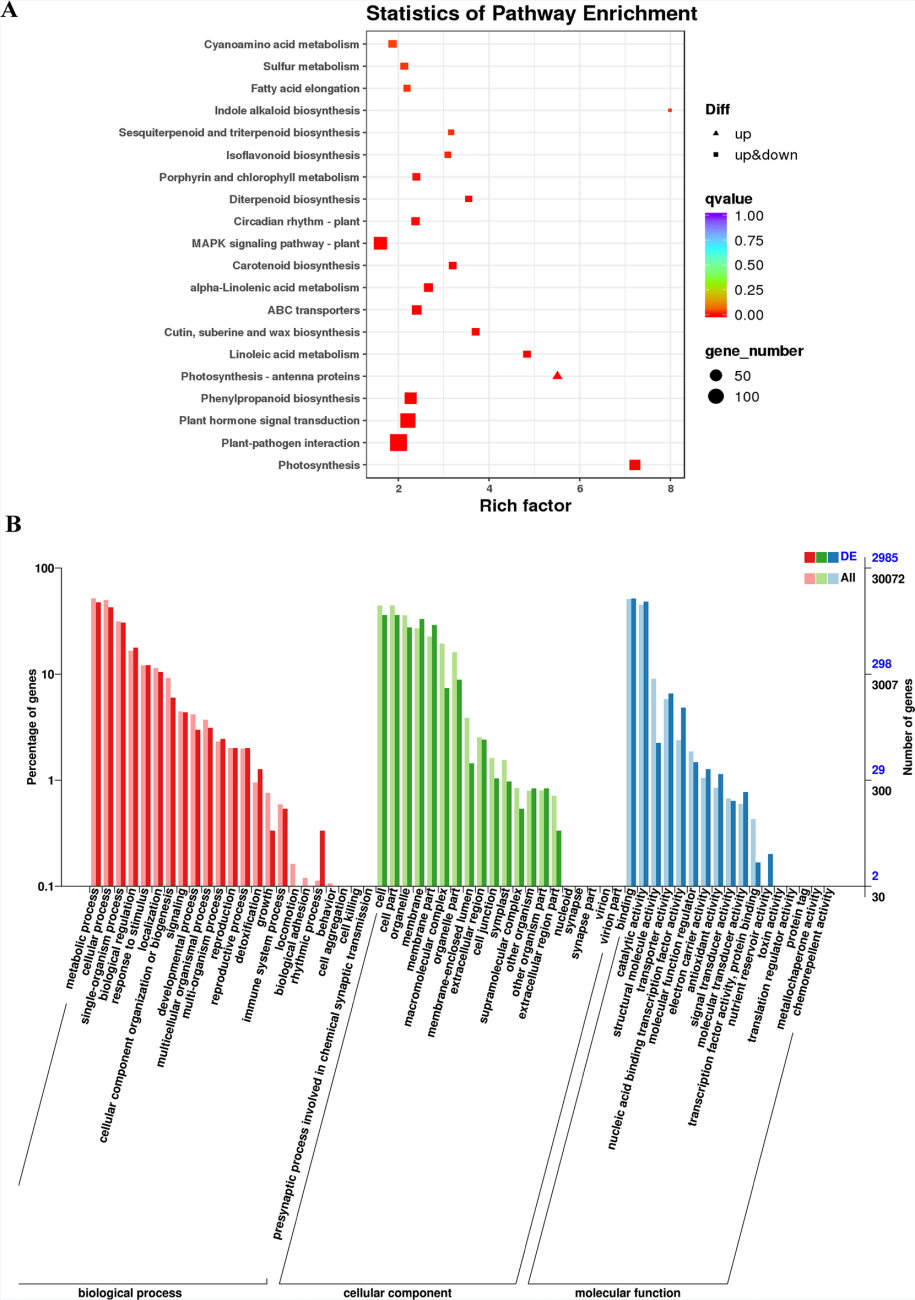
Enrichment analyses. (**A**) The GO enrichment plot of DEGs among samples S1, S2, and S3. (**B**) The KEGG enrichment plot of DEGs among samples S1, S2, and S3.

Combined with our annotation results, we performed KEGG and GO enrichment analysis on the 8361 DEGs we found. The number of DEGs annotated by GO and KEGG database are 2985 and 2024 respectively. The KEGG enrichment results (Fig. 2A) showed that there are significant differences in the gene expression of *G. pentaphyllum* in the redox process and metabolic process of different tissues. By further analyzing the GO enrichment results of DEGs, we found that there are differences in gene expression in the roots, stems and leaves of *G. pentaphyllum* in cellular processes, environmental information processes, metabolism and organic systems (Fig. 2B). Further analysis of these genes, The differences in the expression of these genes are mainly reflected in cellular process, metabolic process, cell, cell part, organelle, binding, and catalytic activity. In the pairwise comparisons of S1vsS2, S1vsS3 and S2vsS3, there was an obvious synthesis of biologically relevant genes for sesquiterpenes, triterpenoids, diterpenoids, phenylpropanoids, isoflavonoids and carotenoids in stems and leaves. Moreover, we also found that the abundance of flavonoids and terpenoids in stems and leaves was consistent with transcript expression levels, while the expression levels of photosynthetic antenna proteins were both upregulated compared with roots. These results indicated that flavonoids and terpenoids were highly expressed in the stems and leaves of *G. pentaphyllum*, resulting in flavonoids and terpenoids being rich in *G. pentaphyllum*.

### Identification of DEGs associated with flavonoid and terpenoid anabolism

Phenylalanine is one of the starting substances in the flavonoid biosynthetic pathway and plays an important role in the synthesis of flavonoids in plants. In this study, we identified a total of 54 DEGs involved in the phenylalanine biosynthetic pathway. Additionally, 8, 3, and 9 DEGs were identified in the flavonoid synthesis, flavonol synthesis and isoflavonoid synthesis pathways, respectively. Comparing the expression levels of these different tissues in* G. pentaphyllum* revealed a noticeable up-regulation of these genes in stems and leaves, including Flavonol Synthase (FLS), Caffeoyl-CoA O-Methyltransferase (HCT), Chalcone Synthase (CHC), Glucosyltransferase (PGT1), Isoflavone 7-O-Glucoside-6''-O-Acetyltransferase (IF7MAT), and Isoflavone/4'-Methoxyisoflavone 2'-Hydroxylase (I2'H). We also identified 3, 11, 9, and 8 candidate DEGs associated with monoterpenoid biosynthesis, triterpenoid biosynthesis, sesquiterpenoid skeleton biosynthesis, and diterpenoid and sesquiterpenoid biosynthesis pathways, respectively. Notably, the expression levels of many DEGs in the stems and leaves of *G. pentaphyllum* were significantly up-regulated, such as 8-Hydroxygeraniol dehydrogenase (10HG) and (-)-α-terpineol synthase in monoterpenoid biosynthesis, Geranylgeranyl pyrophosphate synthase (GA12), Abietadiene oxidase (GA3), Momilactone-A synthase (MAS) in diterpenoid and sesquiterpenoid biosynthesis. Furthermore, we identified 183 transcription factors involved in the regulation of flavonoid and terpenoid biosynthesis in these DEGs that are up-regulated in stems and leaves, including 45 basic helix-loop-helix (bHLH), 17 basic leucine zipper (bZIP), 21 v-myb avian myeloblastosis viral oncogene homolog (myb-related), 24 WRKY, 17 MYB, 53 AP2/ERF, and 6 MADS-MIKC transcription factors.

**Figure 3:**
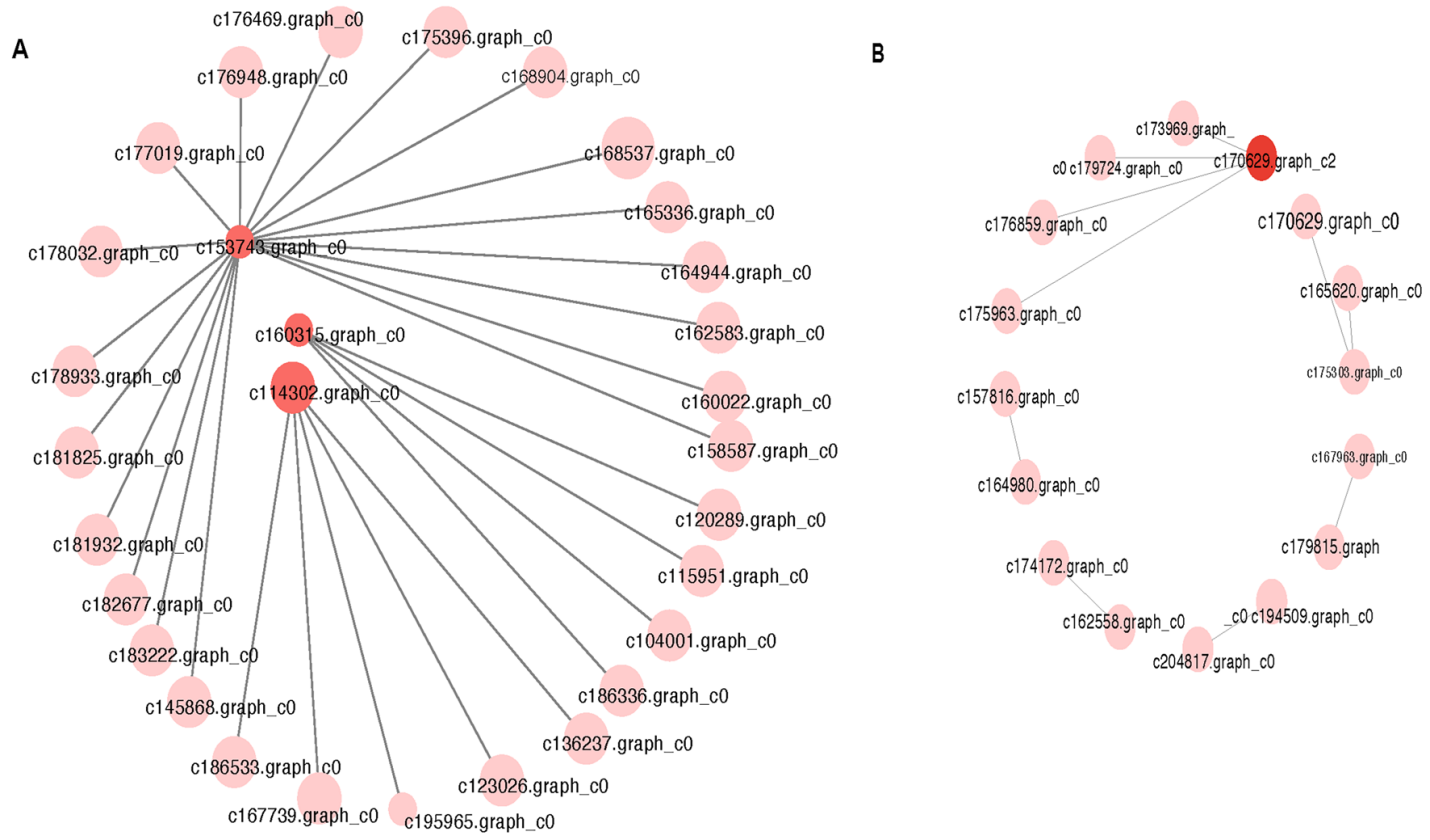
Protein–protein interactions. (**A**) Interaction network among predicted distinct proteins involved in flavonoids biosynthetic pathways. (**B**) Interaction network among predicted unique proteins involved in terpenoids biosynthetic pathways.

Subsequently, we constructed a protein–protein interaction network to analyze the interactions of these genes involved in flavonoids and terpenoids synthesis. Among the 130 DEGs involved in the synthesis of flavonoids(Fig. 3A), 30 DEGs showed strong interactions, including trans-cinnamic acid 4-monooxygenase, dihydroflavonol 4-reductase (DFR), chalcone synthase (CHS), flavonoid 3'-monooxygenase, 2-hydroxyisoflavone synthase, and isoflavone 2'-hydroxylase. Notably, DFR (c114302.graph_c0, c160315.graph_c0, c153743.graph_c0) emerged as a key gene involved in protein interaction. In the 170 DEGs related to terpenoid synthesis, 16 DEGs were closely connected in the network, and 8 of them were key enzymes promoting terpenoid synthesis (Fig. 3B), squalene monooxygenase (SQLE), geranylgeranyl diphosphate synthases (GGPPS) kaurene oxidase (KAO), all-trans-geranylgeranyl diphosphate synthase (SPS), farnesyltransferase beta subunit (FNTB), farnesyltransferase/geranylgeranyl transferase 1 type alpha subunit (FNTA), geranylgeranyl diphosphate synthase II type (GGPS), and trimethyltrienol/dimethylallyl diphosphate isomerase, respectively. And farnesyl diphosphate synthase (FDPS, c170629.graph_c2) emerged as a core gene in the network.

### Gene co-expression related genes networks

**Figure 4:**
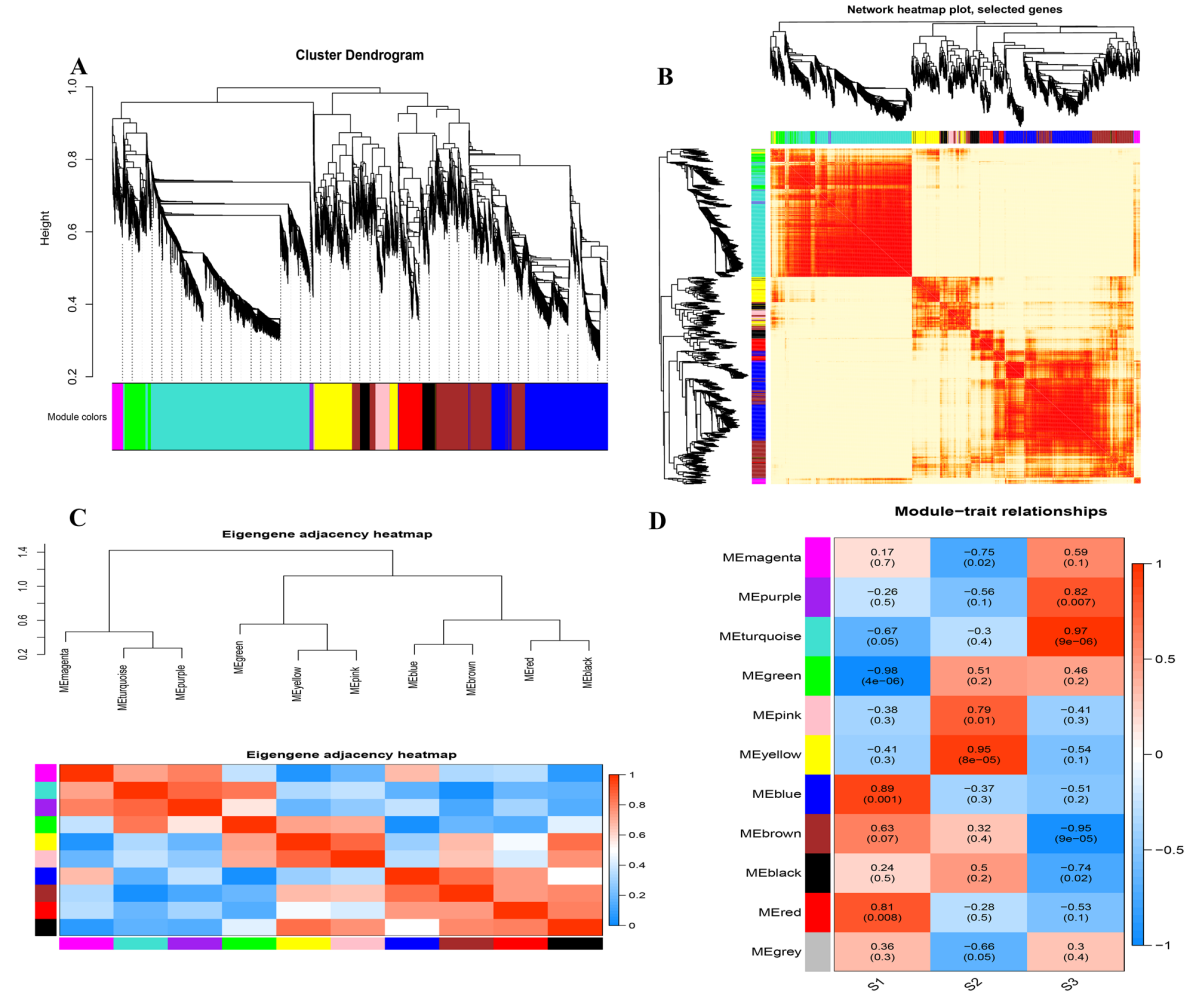
Weighted gene co-expression network analysis (WGCNA) of the identified genes in *G. pentaphyllum.* (**A**) Gene tree diagram obtained by overlapping clustering based on consensus topology. Each branch constitutes a module, and each leaf represents a gene. Each row of color represents a color-coded module containing a set of highly interconnected genes. (**B**) Heatmap plot of topological overlap in the gene network. Darker squares along the diagonal correspond to modules. (**C**) The correlation among the physiological indicators of the intrinsic genes of the module and the samples. (**D**) The correlation coefficient among the physiological indicators of the intrinsic genes of the module and the samples.

We constructed a co-expression network with the results of total flavonoids and terpenoids in *G. pentaphyllum* and RNA-seq data to analyze the WGCNA modules relevant to terpenoid and flavonoid biosynthesis in various tissues of *G. pentaphyllum*. The WGCNA analysis showed that the 8361 DEGs were divided into 11 different modules (Fig. 4A). It is worth noting that the accumulation patterns of terpenoids and flavonoids in the two modules of turquoise and purple are greatly correlated, including two modules of 2747 and 56 single genes, and the absolute correlation coefficients (ACC) are all more than 0.8 (*p* value ≤ 0.01). Then we plotted gene heatmaps and bar graphs across all samples to particularly detect the transcriptional expression outlines of these modules, where the expression moduli of signature genes in turquoise and purple were highest in S3 samples (Fig. 4B–D).

**Figure 5:**
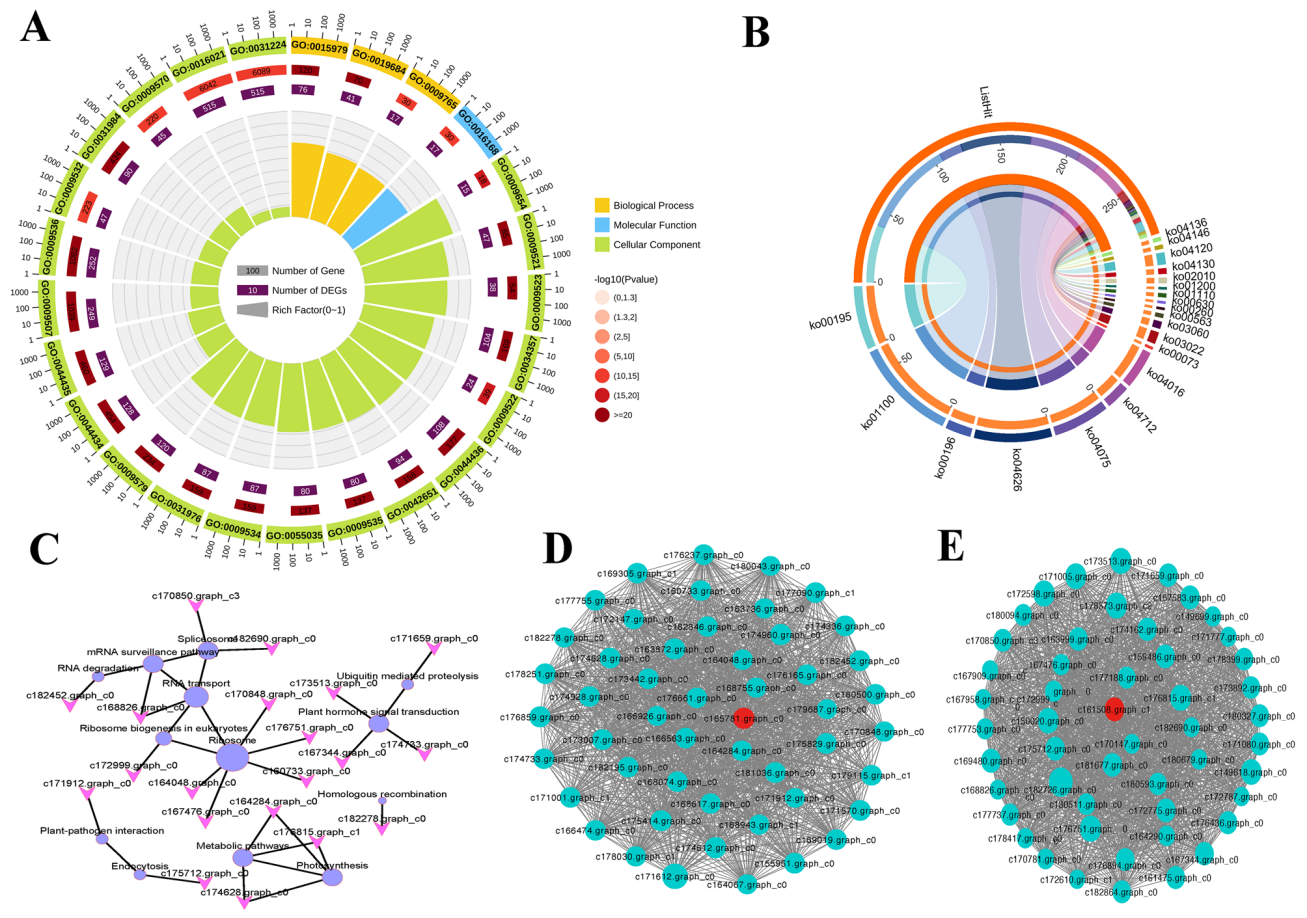
Enrichment analyses and gene networks of WGCNA modules. (**A**) GO circle plot showing gene annotation enrichment analysis. (**B**) KEGG chart showing gene annotation enrichment analysis. (**C**) Hub genes. (**H**) Cytoscape represents the top 50 co-expressed genes in the turquoise module. (**I**) Cytoscape represents the top 50 co-expressed genes in the purple module.

To further analyze these genes related to terpenoid and flavonoid biosynthesis, we performed KEGG and GO enrichment analysis on the genes of these modules. The results of GO enrichment (Fig. 5A) analysis showed that genes related to terpenoid and flavonoid biosynthesis were mainly concentrated in cellular processes. The KEGG pathway (Fig. 5B) is mainly involved in photosynthesis, metabolic pathways, plant hormone signal transduction, and biosynthesis of secondary metabolites, which is consistent with the previous results of this study. Hub genes are commonly used to analyze genes in gene co-expression networks, and they are highly connected in this study. A total of 24 unigenes were screened out using Cytohubba's 11 topological analysis methods^57^, 13 of which were protein-coding genes (Fig. 5C). These genes play a key role in the gene co-expression network, participating in information transmission, regulating network stability, and functional regulation.

Grounded on the connectivity (KME) values of eigengenes, the top 50 genes in the turquoise and purple modules were selected for protein interaction analysis. We analyzed the relationship of protein interactions in green and purple modules using Cytoscape_v.3.7.1, the highlighted gene encoding the uncharacterized protein LOC111481776 (c165781.graph_c0) (Fig. 5D) showed the greatest KME value and most close accompanied with other node genes in the turquoise module, and the gene encoding UDP-glucose 4-epimerase GEPI48 (c161508. graph_c1) (Fig. 5E) was a core member of the purple module.

### Enrichment analysis of the DAMs

**Figure 6:**
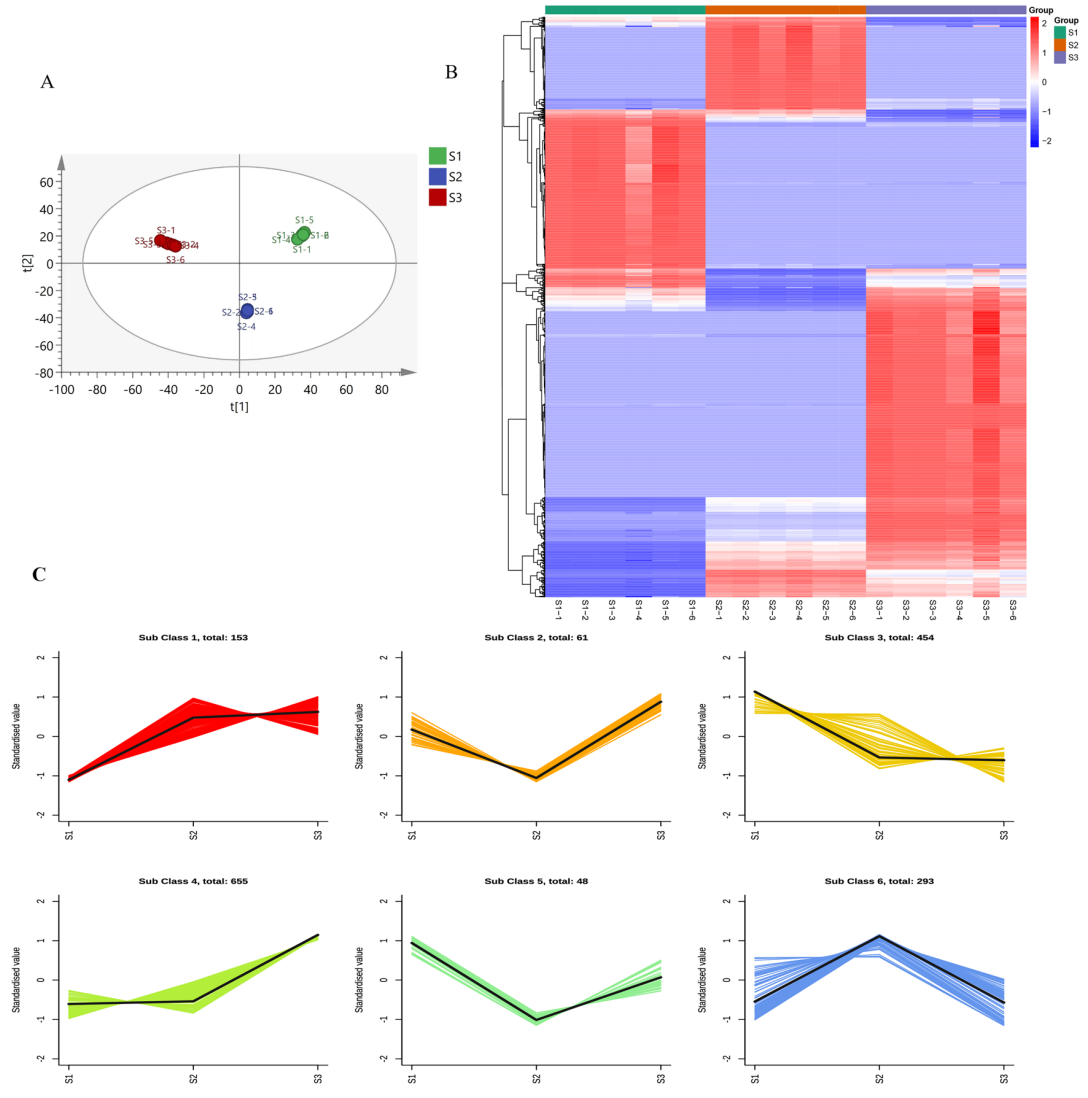
Enrichment Analysis of the differentially accumulated metabolites (DAMs). (**A**) PCA score graph. (**B**) Clustering heatmap. (**C**) K-means analysis of metabolites.

We conducted an extensive and targeted metabolomic analysis of th *G. pentaphyllum*.to construct its metabolic profile. A total of 1,665 metabolites were detected in the three tissues of C. cinquefoil, mainly including flavonoids, phenolic acids and terpenoids. And secondary metabolites account for a large proportion of known metabolites, indicating that *G. pentaphyllum* has strong secondary metabolism activity. The results of PCA analysis (Fig. 6A) showed that the root, stem and leaf of *G. pentaphyllum* were divided into three distinct clusters which indicated that there were significant differences in the metabolism of the three tissues. To further explore the differences in metabolites of *G. pentaphyllum* root, stem and leaf, we used OPLS-DA to conduct pound-two comparison of root, stem and leaf (Figure S1), and the results further proved that there were significant metabolic differences among different tissues. This study also further analyzed the metabolic differences amog different tissues from the perspective of metabolite content. Figure 6B showed that the metabolite content in the roots, stems and leaves of *G. pentaphyllum* has a large difference, while there is almost no difference in the metabolite content in the same tissue which also shows that the metabolomics results of this study have strong reliability. Then we performed average enrichment analysis on 1665 metabolites (Fig. 6C) and divided them into 6 clusters based on the relative abundance of metabolites. The largest cluster, Cluster 4, contains 655 metabolites and shows a significant decrease in S1 and S2, while a significant increase in S3.

### WGCNA related metabolic networks

**Figure 7:**
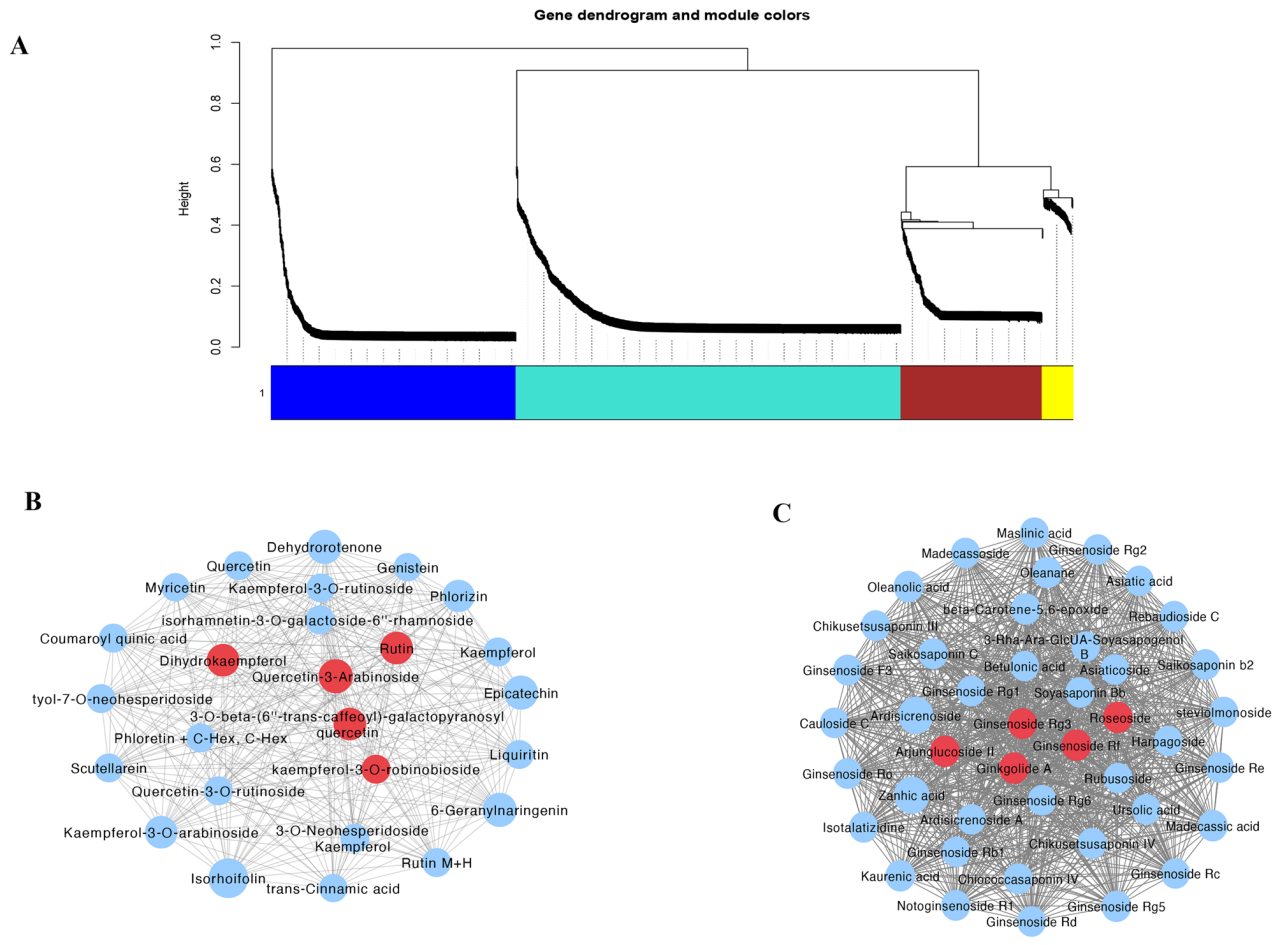
The WGCNA co-expression network analysis of DAMs. (**A**) Gene tree diagram obtained by overlapping clustering based on consensus topology. Each branch constitutes a module, and each leaf represents a gene. Each row of color represents a color-coded module containing a set of greatly connected genes. (**B**) Cytoscape showing flavonoid metabolites in the turquoise module. (**C**) Cytoscape shows terpenoid metabolites in the turquoise module.

The results of WGCNA analysis showed that 1665 metabolites were divided into 4 different modules, among which the accumulation patterns of terpenoids and flavonoids in turquoise module were very correlated with a total of 799 metabolites (Fig. 7A). In addition, 32 flavonoids and 43 terpenoid metabolites in the turquoise module were selected based on the connectivity values of the characteristic genes to generate co-expression subnetworks visualized using Cytoscape to find significant contributing metabolites. In flavonoid synthesis, Rutin, dihydrokaempferol, 3-O-β-(6''-trans-caffeoyl)-galactopyranosyl quercetin, Quercetin-3-Arabinoside have the highest KME value (Fig. 7B) and is associated with turquoise, but also with other nodes Metabolites are most closely related. In the synthesis of terpenoids (Fig. 7C), the ajunoglucoside II, ginsenoside Rg3, ginsenoside Rf, rosaponin, and ginkgolide modules are most closely related to other node metabolites and belong to the terpene core members of the turquoise module.

### Joint analysis of DEGs and DAMs

**Figure 8:**
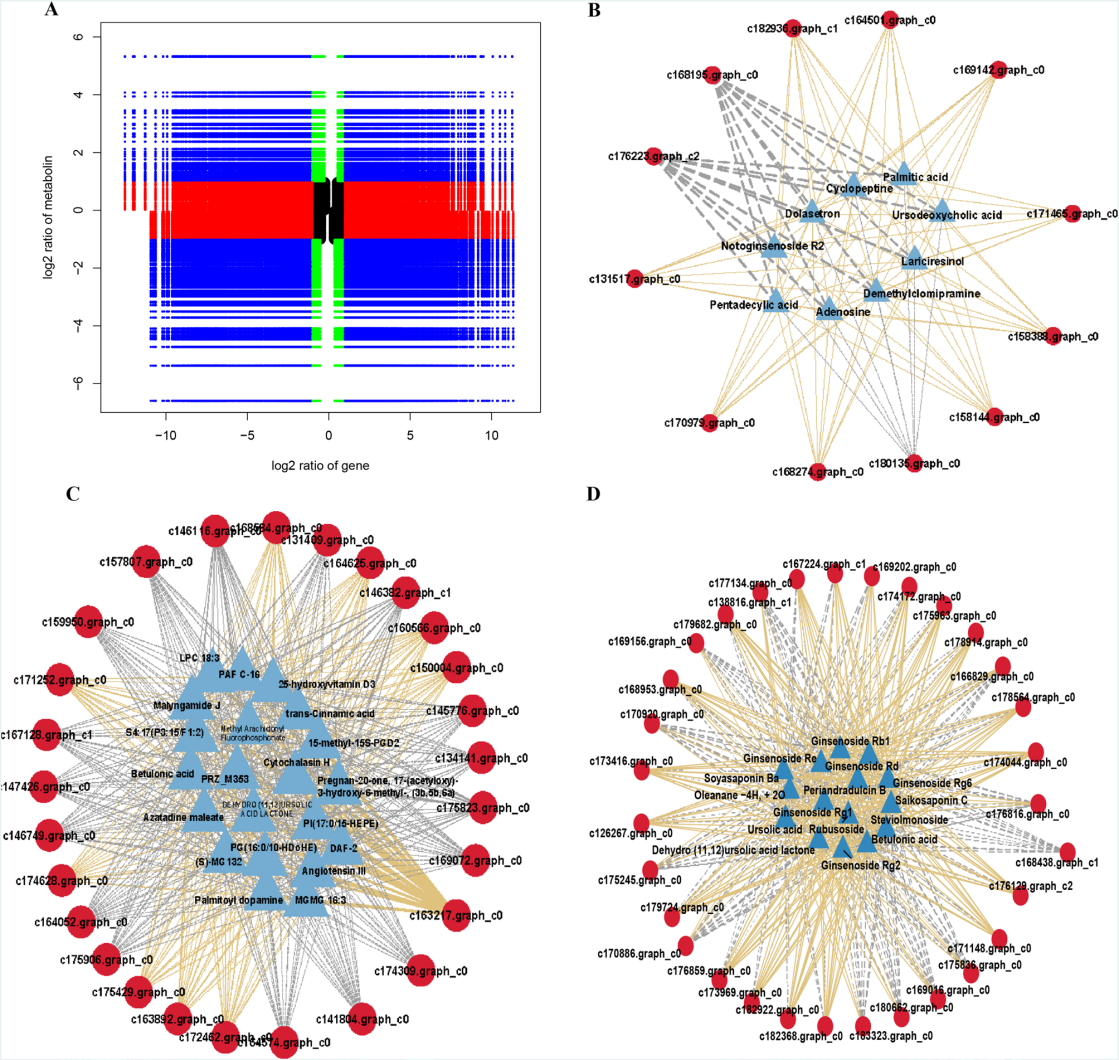
Bioinformatics analysis of matched DEGs and DAMs**.** (**A**) A nine-quadrant plot of distinctly expressed genes and distinctly accumulated metabolite correlations. (**B**) A connection network of flavonoid synthesis among different expressed genes (red ovals) and differentially accumulated metabolites (blue triangles). (**C**) A connection network of flavonoid synthesis among different expressed genes (red ovals) and of flavonoid synthesis differentially accumulated metabolites (blue triangles). (**D**) A connection network ofterpenoid synthesis among differential expressed genes (red ovals) and differentially accumulated metabolites (blue triangles).

Our previous results have shown that both DEGs and DAMs are involved in the flavonoids and terpenoids biosynthetic pathways which indicate that *G. pentaphyllum* has strong flavonoid and terpenoid metabolic activity. To further explore whether these DEGs and DAMs are involved in the same ketone and terpene biosynthetic pathways, we performed correlation analysis on each group of DEGs and DAMs. The results showed that many metabolites were negatively or positively regulated by multiple genes (Fig. 8A). The DEM and DEG in quadrants 3 and 7 are positively correlated, while the DEM and DEG in quadrants 1 and 9 are negatively correlated.

In the synthesis of flavonoids, we screened out a total of 250 DEGs that were significantly related to 30 DAMs in *G. pentaphyllum*. For example, betulinic acid was significantly correlated with 27 DEGs (10 positive, 17 negative), and dehydro(11, 12) ursolic acid was significantly correlated with 27 DEGs (10 positive, 17 negative). Interestingly, we found two networks of metabolite and gene interactions involved in flavonoid synthesis in *G. pentaphyllum*. The DEG was identified as c169142.graph_c0, annotated as flavonoid dehydrogenase and notoginseng saponin R2 (Fig. 8B), positive correlations with nine metabolites such as palmitic acid, adenosine, dolasetron, ursodeoxycholic acid, pentadecanoic acid, Perianandradulcin B, cyclic peptide base. The DEG-tagged c172462.graph_c0 was annotated as NMT and was positively correlated with phosphoethanolamine methyltransferase, trans-cinnamic acid, betulinic acid, 9-fluorenone, cytochalasin H, 25-hydroxycholecalciferol (Fig. 8C). These results suggest that *G. pentaphyllum* has a sophisticated administrative mechanism between changes in flavonoid metabolite accumulation and gene expression abundance.

However, DEGs significantly related to terpenoid accumulation are rarely found. To better understand the molecular mechanism of terpenoid accumulation, we further screened terpenoid-related DEGs and DAMs. Correlation analysis results showed that rubusoside was significantly correlated with 31 DEGs (19 positive, 12 negative) in terpene metabolites, including GERD, 10HGO, GA12, chlP, bchP, FDPS, GA2ox, KAO, SPS, SQLE, ERG1, FDPS, MAS. The DEG is marked as c182368.graph_c0 and annotated as FDPS (Fig. 8D), and is positively correlated with 11 metabolites including betulinic acid, ginsenoside, Periandradulcin B, Rubusoside, Saikosaponin C, and Steviolmonoside.

### Validation of differential expression by qRT-PCR

**Figure 9:**
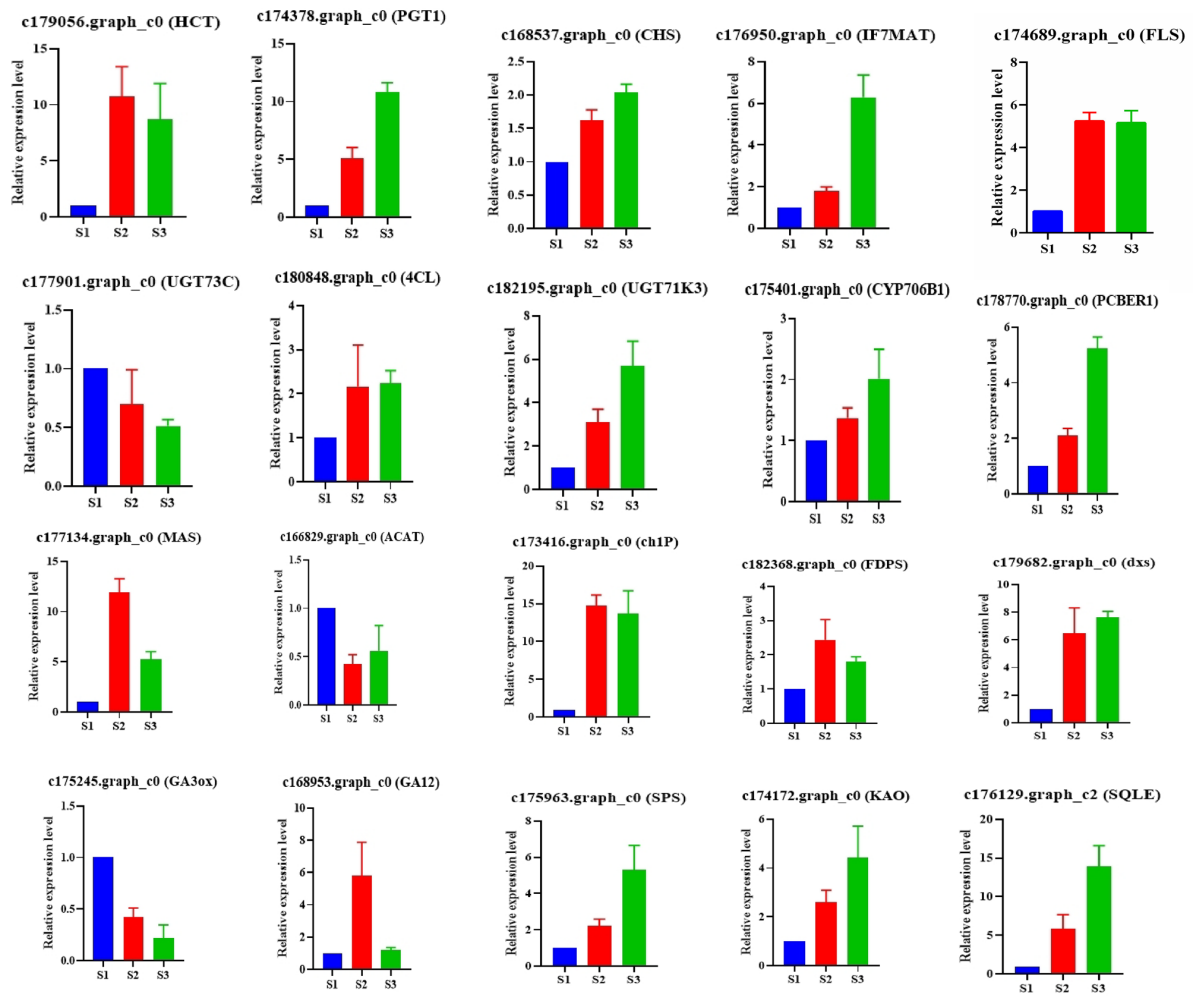
Differential expression of flavonoid and terpenoid synthesis was verified by qRT-PCR.

To verify the accuracy of the transcriptome and metabolome data analysis results, we selected 10 representative DEGs in the mRNA involved in flavonoid and terpenoid anabolism respectively for qRT-PCR detection (Fig. 9). The 20 representative DEGs selected in this study can all be detected in the three tissue of *G. pentaphyllum*, which further demonstrates the accuracy of the analysis results of this study.

## Discussion

*G. pentaphyllum* contains a variety of biologically active substances, such as flavonoids and terpenoids. However, some studies on the accumulation and regulation of flavonoids and terpenoids in *G. pentaphyllum* are still based only on transcriptome sequencing, which cannot provide a complete metabolic picture of biological activities. This study used RNA-seq and UHPLC-QTOF-MS technology to preliminarily analyze the dynamic changes in the nutritional components of *G. pentaphyllum* tissues and their possible molecular mechanisms. In our study, a total of 94,850 unigenes and 1665 metabolites were assembled. The highest number of DEGs was found in the comparison of stem and leaf tissues (S3 and S1), and the highest number of DAMs were also detected, which means that more changes in biological processes may occur in *G. pentaphyllum* leaves.

### Flavonoid biosynthesis

**Figure 10:**
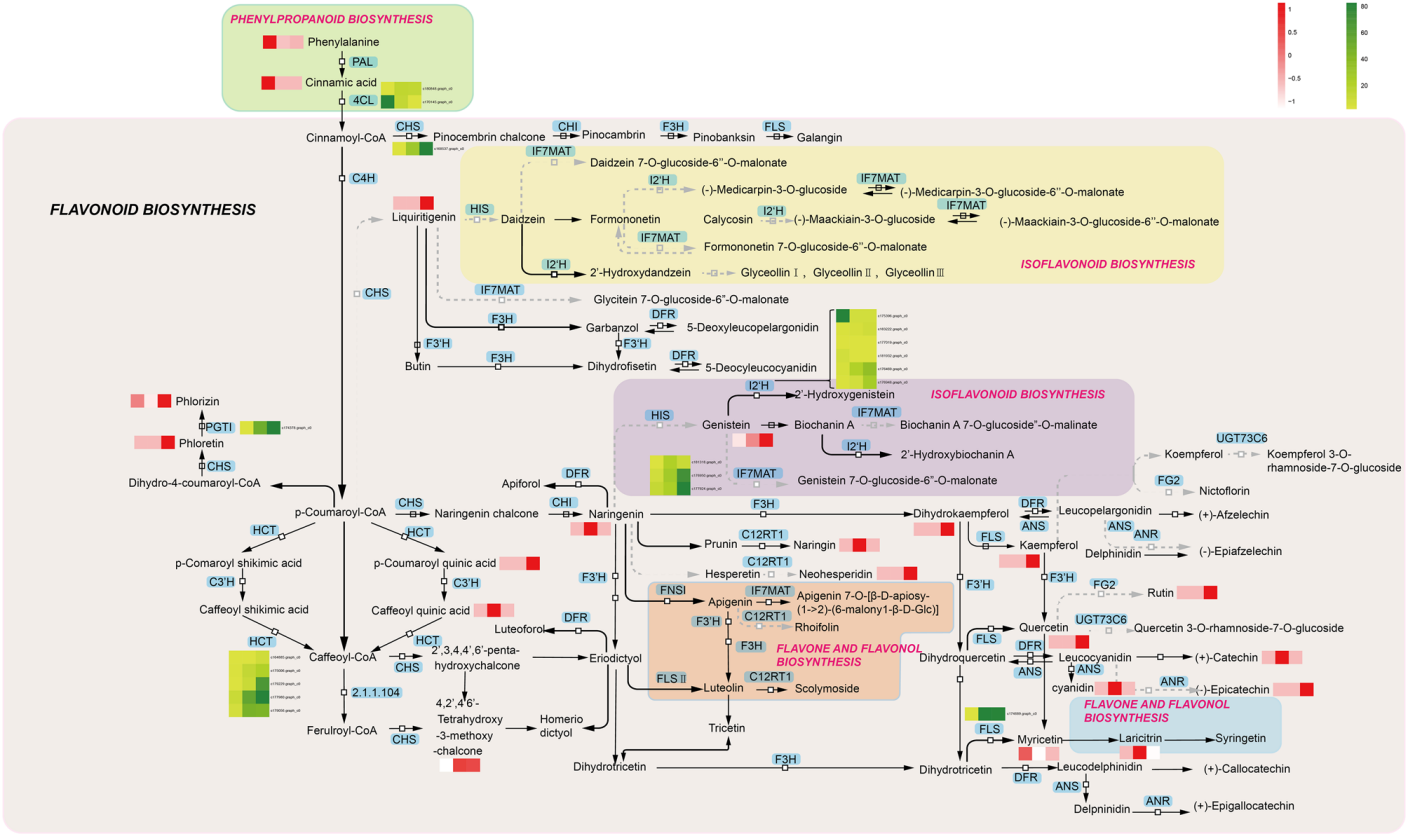
Visualization of protein and transcript expression in a biochemical pathway map related to flavonoid biosynthesis in *G. pentaphyllum.*

Flavonoids^58^ are a class of polyphenol secondary metabolites widely present in sperm, including flavonols, flavonoids, 3-flavanols, isoflavones, flavonoids and anthocyanins. Many studies have shown that flavonoids have medical properties such as antioxidant, anti-inflammatory and anti-tumor activity, vasoactive activity, estrogenic activity and other biological applications^59–61^. Flavonoids are products of phenylpropanoid metabolism and are considered to be the bridge between primary and secondary metabolism. This regulatory network starts with phenylalanine and uses p-coumaroyl-CoA as the precursor (Fig. 10)^62^. It is catalyzed by PAL and 4CL at key positions to control carbon migration to generate phenylalanine and cinnamic acid, and further enters into the synthesis of flavonoids. In this study, Fig. 5A shows the candidate enzymes annotated at different expression levels of mRNA. Several members may be identified as the same enzyme, perhaps because they are alternative splicing^63^ or specific gene families.

The three aromatic rings produced by chalcone synthase (CHS) form the basic skeleton of all flavonoids^64^, and only one such corresponding unigene (c168537.graph_c0) was identified in our dataset which is related to the roots of *G. pentaphyllum*, and expression is upregulated in *G. pentaphyllum* leaves. We also detected a small amount of naringenin in the stems of *G. pentaphyllum*. Further formation of dihydroflavonols, such as dihydrokaempferol^65^, accumulates in *G. pentaphyllum* leaves with the participation of naringenin 3-dioxygenase (F3H). FLS is an immobilized enzyme that competes with DFR at critical branch points. Therefore, it can be used to convert dihydrokaempferol, dihydroquercetin, and dihydromyricetin into aglycones (flavonols). A unigene (c174689.graph_c0) encoding this portal enzyme was identified, and its expression level was highly upregulated, consistent with the synthesis of quercetin and myricetin among the metabolites. And under the action of 2-hydroxyisoflavone synthase (HIS, c136237.graph_c0)^66^, the naringenin metabolic pathway flows to the isoflavone biosynthetic pathway to generate cytisin, and high expression was detected in *G. pentaphyllum* leaves.

Under the action of flavanone 7-O-glucoside 2''-O-β-L-rhamnosyltransferase (C12RT1)^67^, the metabolic pathway of naringenin flows to the flavonoid and flavonol biosynthesis pathway to produce naringin and Isorhoifolin, which are detected and highly expressed in the stems and leaves of *G. pentaphyllum*. Anthocyanin reductase (ANR) and anthocyanin synthase (ANS)^68^ are key downstream enzymes in the phenotypic and non-phenotypic and biosynthesis of catechins. Their main function in *G. pentaphyllum* is to convert colorless anthocyanins into cyanidin^69^ and is absorbed by the root tissue of *G. pentaphyllum*, and further generates ( +)-epicatechin and ( +)-catechin. Quercetin can also be further converted to rutin by flavonol-3-O-glucoside L-rhamnosyltransferase (FG2, c169543.graph_c0). In the flavonoid and flavonol biosynthetic pathway, myricetin can be converted into lariculin through AOMT enzyme.

In addition, shikimate O-hydroxycinnamyl transferase (HCT) is considered a reversible enzyme^70^, and inhibiting the expression of HCT can lead to the accumulation of flavonoids. In this study, the five structural DEGs encoding HCT (c164885.graph_c0, c175006.graph_c0, c177980.graph_c0, c179056.graph_c0, c179229.graph_c0) were abundantly expressed in leaves, which were also related to the content of flavonoids in the leaves. At the metabolome and transcriptome levels, these positive regulatory enzymes involved in the synthesis of flavonoids are significantly expressed in *G. pentaphyllum* leaves, confirming that flavonoid metabolic compounds such as quercetin, isorhamnetin, and kaempferol accumulate in the leaves.

### Terpenoid biosynthesis

Plant-derived terpenoids^71^ are natural products with the most structural changes in plants and have a wide range of physiological functions. Gibberellins, abscisic acid, insect larvae hormones, carotenoids and chlorophyll are important photosynthetic pigments in plants^72^. Plastoquinone and ubiquinone are important electron transmitters in the photosynthetic chain and respiratory chain^73^. In nature, terpenoids are widely found in various plants, and these plants all have physiological activities. For example, amaranth has an anthelmintic effect^74^, artemisinin has an antimalarial effect^75^, andrographolide has an antibacterial effect^76^. The synthesis of terpenoids in plants can occur either end-to-end via isoprene or via isoprene ring formation^77^. Isoprene first needs to be converted into isopentenyl pyrophosphate (IPP) and dimethylallyl pyrophosphate (DMAPP) through activation. The two pathways for the synthesis of IPP and DMAPP in vivo are mevalonate dependent (MVA) pathway formed by triacetyl-CoA (Acetyl-CoA) and methylerythritol (MEP) pathway formed by pyruvate or glycerol 3-phosphate pathway^78^. The choice of different pathways depends on the species of organism and the subcellular location of the synthesized products.

**Figure 11:**
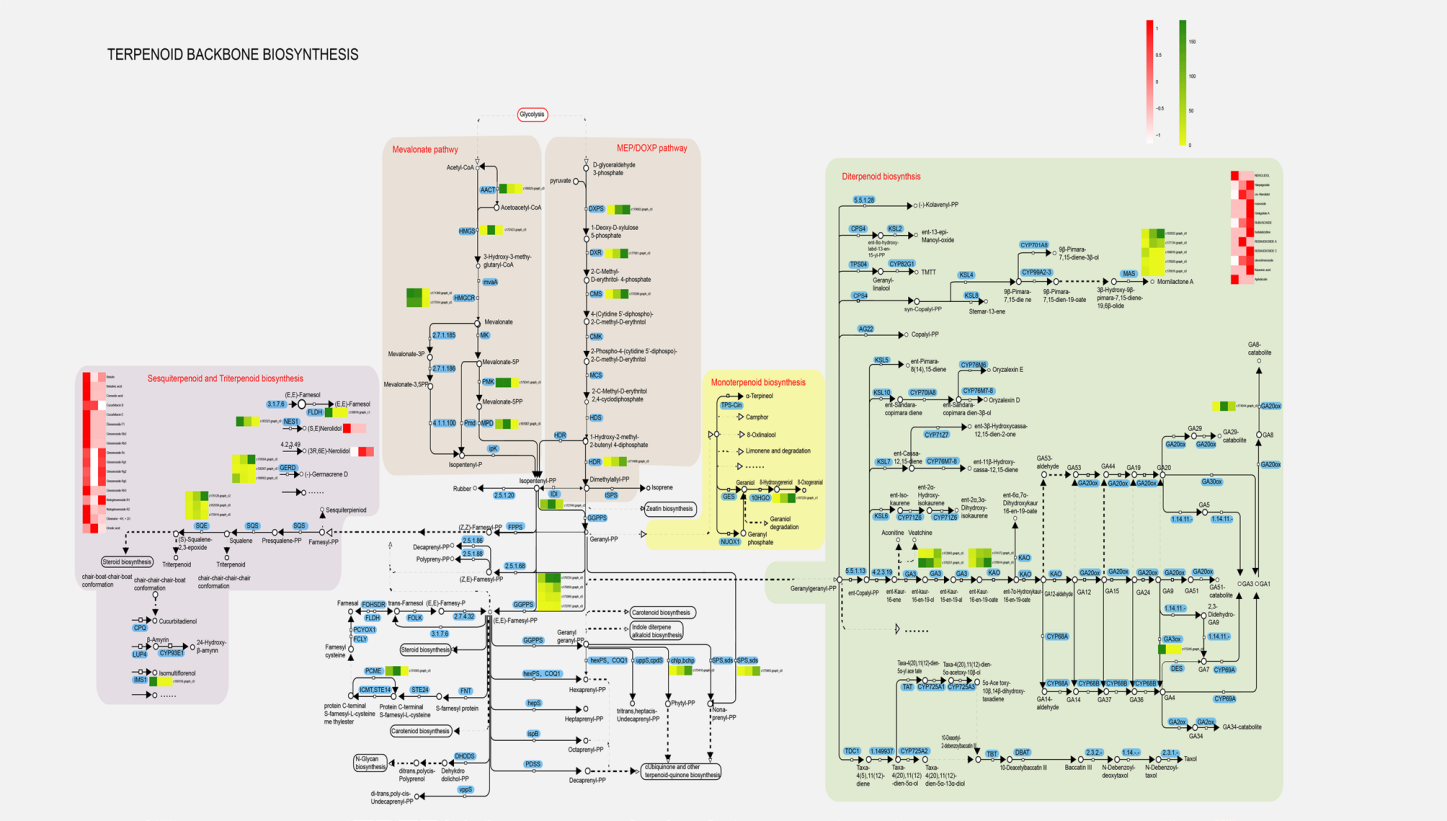
Visualization of protein and transcript expression in a biochemical pathway map related to terpenoid biosynthesis in *G. pentaphyllum.*

Hydroxymethylglutaryl-CoA reductase (HMGCR)^79^ catalyzes the irreversible production of MVA from 3-hydroxy-3-methylglutaryl-CoA (HMG-CoA) (Fig. 11). 3-Hydroxy-3-methylglutaryl-CoA is a sterol and metabolite. It is the first rate-limiting enzyme in the pentadiene biosynthesis pathway and a key regulatory point in the metabolism of terpenoids in animals and fungi. HMGCR plays a key role in changes in "carbon flux", determining the ratio of various terpenoid end products. In the comparison of three tissues in this study, the expression of three unigenes of HMGCR (c172423.graph_c0, c171369.graph_c0; c177914.graph_c0) was reduced in *G. pentaphylla* root tissue, while phosphomyvalonate kinase (PMK) was expressed in stems and leaves. , c178341.graph_c0) and mevalonate diphosphate decarboxylase (MPD, c181007.graph_c0) expression were generally reduced, indicating that the MVA pathway mainly plays a role in *G. pentaphylla*.

The MEP pathway uses pyruvate as the raw material for glyceraldehyde 3-phosphate^80^, which is polymerized under the action of 1-deoxyxylose-5-phosphate synthase (DXPS). DXP is then catalyzed by 1-deoxyxylose-5-phosphate reductoisomerase (DXR)^81^ to form MEP, and then undergoes phosphorylation, cyclization and other steps to generate IPP, thereby condensing into terpenoids such as monoterpenes and diterpenes. 1-Deoxyxylose-5-phosphate synthase is the first key enzyme in the MEP biosynthetic pathway and a potential new antibiotic, antimalarial, and herbicide. It is highly expressed in *G. pentaphylla* stems and leaves (c179682.graph_c0), suggesting that the MEP pathway may play a role in stems and leaves.

IPP is the core precursor for the synthesis of all terpenoids^82^. Prenyltransferase The basic building block of the MVA and MEP terpenoid biosynthetic pathways is the chain extension reaction, which is catalyzed by prenyltransferase (diphosphate synthase)^83^. It catalyzes IPP and its isomers to generate GPP, and then sequentially adds different numbers of IPP units to GPP to obtain the corresponding sesquiterpenes and diterpenes. GGPPS (c173969.graph_c0, c176859.graph_c0, c179724.graph_c0, c182368.graph_c0, c172787.graph_c0) is highly expressed in the stems and leaves of *G. pentaphyllum*. These enzymes are key enzymes for the synthesis of diterpenes, tetraterpenes and polyterpenes and this one result is consistent with the results of *G. pentaphylla* metabolites.

## Conclusion

This study used a combination of RNA-seq transcriptome analysis and metabolomics technology to study the dynamic changes of substance accumulation in different tissues of *G. pentaphyllum*. Through systematic analysis of transcriptome and metabolome data of *G. pentaphyllum*, we detected a total of 50,323 gene sequences and 1665 metabolites. On this basis, we paid special attention to the biosynthetic and metabolic processes of flavonoids and terpenoids in *G. pentaphyllum* and explored the possible regulatory mechanisms. By combining the expression profiles of genes and transcription factors, as well as the contents of corresponding compounds, we revealed the strong synthesis ability of flavonoids in *G. pentaphyllum* leaf tissues, while terpenoids showed dynamic changes in different tissue parts. This comprehensive study provides insights into the comprehensive understanding of the dynamic distribution of genes and metabolites in different tissues of *G. pentaphyllum*. Further exploration of key genes related to the synthesis pathways of flavonoids and terpenoids will help promote the application of *G. pentaphyllum* in model organisms and provide scientific support for its potential value in medicine. This research data on *G. pentaphyllum* not only provides an in-depth understanding of its medicinal value, but also provides useful references and ideas for the research of other medicinal plants. By integrating transcriptome and metabolome information, we provide a scientific basis for further exploring the regulatory mechanisms of plant natural product synthesis pathways and drug development.

### Supplementary Information

Supplementary Legends.

Supplementary Figure 1.

Supplementary Table 1.

Supplementary Table 2.

Supplementary Table 3.

## Data Availability

Sequence data that support the findings of this study have been deposited in the European Nucleotide Archive with the primary accession code PRJNA1054609.
