# Supplementary material for: Integrative metabolomic and transcriptomic analyses reveals the accumulation patterns of key metabolites associated with flavonoids and terpenoids of Gynostemma pentaphyllum (Thunb.) Makino
Source: Sci Rep. 2024 Apr 15;14:8644. doi: 10.1038/s41598-024-57716-5 (PMC11018608; doi:10.1038/s41598-024-57716-5)
Supplement: Supplementary file 1 — Supplementary Legends. [file 41598_2024_57716_MOESM1_ESM.docx]

**Supplementary Materials:**

Table S1 The evaluation statistics of 9 raw transcript datasets of *G. pentaphyllum*. S1, S2, S3 are the roots group, stems group and leaves group, respectively

Table S2 The Sequence length and basic information of transcriptome assembly results of *G. pentaphyllum*.

Table S3 The annotated results of the *G. pentaphyllum* assembly of unigenes in eight databases. COG: Cluster of Protein Homology Groups, Swiss-Prot: Human Annotated and Reviewed Protein Sequence Database, Pfam: Protein Families, KEGG: Kyoto Encyclopedia of Genes and Genomes, NR: NCBI Non-redundant Protein Sequences, GO: Gene Ontology.

Figure S1. OPLS-DA scatter plot and permutation test plot of S1 vs S2 (Lift), S1 vs S3 (Middle) and S2 vs S3 (Right).
