# Supplementary figures and images for: Integrative metabolomic and transcriptomic analyses reveals the accumulation patterns of key metabolites associated with flavonoids and terpenoids of Gynostemma pentaphyllum (Thunb.) Makino
Source: Sci Rep. 2024 Apr 15;14:8644. doi: 10.1038/s41598-024-57716-5 (PMC11018608; doi:10.1038/s41598-024-57716-5)

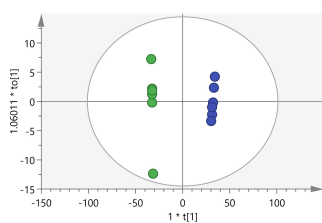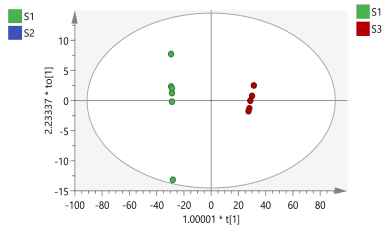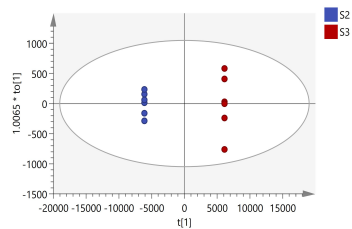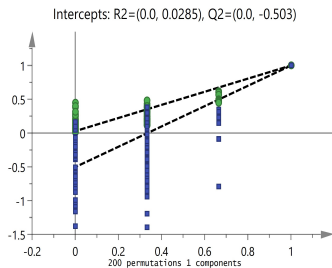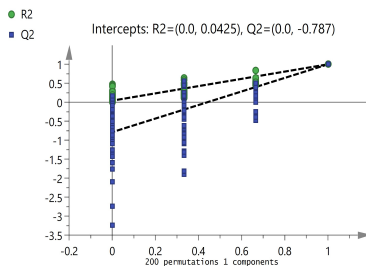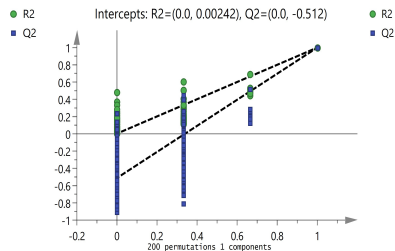

Supplement: Supplementary file 2 — Supplementary Figure 1. [file 41598_2024_57716_MOESM2_ESM.pdf]
